# The impact of COVID-19 on kidney transplant activities in Brazil: a descriptive study

**DOI:** 10.1590/1516-3180.2021.0956.R1.29042022

**Published:** 2022-08-29

**Authors:** Reginaldo Passoni, Bruno Gadonski, Ariana Rodrigues da Silva Carvalho, Tainá Veras de Sanders Freitas, Luis Alberto Batista Peres

**Affiliations:** I RN, MSc. Nurse, Department of Nursing, Hospital Universitário, Universidade Estadual do Oeste do Paraná (UNIOSTE), Cascavel (PR), Brazil.; II Medicine Student, Center for Medical and Pharmaceutical Sciences, Universidade Estadual do Oeste do Paraná (UNIOSTE), Cascavel (PR), Brazil.; III RN, PhD. Professor, Postgraduate Program in Biosciences and Health, Center for Biological and Health Sciences, Universidade Estadual do Oeste do Paraná (UNIOSTE), Cascavel (PR), Brazil.; IV MD, PhD. Professor, Departament of Clinical Medicine, Universidade Federal do Ceará (UFC), Fortaleza (CE), Brazil.; V MD, PhD. Professor, Postgraduate Program in Biosciences and Health, Center for Biological and Health Sciences, Universidade Estadual do Oeste do Paraná (UNIOSTE), Cascavel (PR), Brazil.

**Keywords:** COVID-19, Kidney transplantation, Tissue and organ procurement, Epidemiology, Organ transplantation, Transplant, Tissue donor, Pandemic, Coronavirus infection

## Abstract

**BACKGROUND:**

The coronavirus 19 (COVID-19) pandemic has reached services, systems, and world society. Despite its certified efficiency, the Brazilian National Transplant System is not exempt from the side effects of COVID-19.

**OBJECTIVE:**

To compare kidney transplantation activity registered in Brazil between the pandemic (2020) and pre-pandemic (2019) periods.

**DESIGN AND SETTING:**

A descriptive study was conducted in March 2021. The annual reports of the Brazilian Transplantation Registry for 2019 and 2020 were included in this study.

**METHODS:**

We conducted a descriptive study of kidney transplant activity in Brazil in 2019 and 2020.

**RESULTS:**

A 23.9% decrease in kidney transplants per million population was observed during the pandemic period (22.9 in 2020 versus 30.1 in 2019). Kidney transplants with a living donor (-58.8%) and in the North Region (-79.5%) experienced the greatest declines. The pandemic waiting list increased by 6.8%, and deaths during the waiting period increased by 36.8%. The number of patients on the waiting list and transplant teams decreased by 31.3% and 9.5%, respectively.

**CONCLUSION:**

The COVID-19 pandemic drastically affected Brazil and had a significant negative impact on KT activities in the country.

## INTRODUCTION

After the outbreak in China, severe acute respiratory syndrome coronavirus 2 (SARS-CoV-2) infection has rapidly spread to other countries and continents. The pandemic of coronavirus disease 19 (COVID-19) has reached services, systems, and global society. The first case of COVID-19 in Brazil was confirmed in mid-February 2020, and within days the number of cases increased dramatically.^1^ The Brazilian epicenter of COVID-19 was in the southern region, in the city of São Paulo. However, several cities in the north and northeast of the country also experienced alarmingly high numbers of cases and deaths in the first months of the disease's registration.^1^ the side effects of COVID-19 go beyond devastating damage to human health and undermine the structure, processes, and outcomes of a nation's services and systems. Despite its certified efficiency, the Brazilian National Transplant System was not exempt from the side effects of COVID-19, and the first negative impact of the pandemic on the organ donation-transplant process in Brazil was observed in the first quarter of 2020.^2^

Compared with the same period in 2019, there was a 9.5% decrease in heart transplants and a 30% and 22.2% decrease in the number of living kidney and liver donors, respectively, in the first quarter of 2020. Of the 27 Brazilian states, only six (22.2%) performed pancreas transplants, and one state in the Northeast saw a 27% decrease in liver transplants. Additionally, there was a significant decrease in the number of transplants for all solid organs in March 2020 compared with January and February ^2^ which corresponds to the period when records of COVID-19 infections began in the country, resulting in an exponential increase in the number of confirmed cases. Alternatively, we observed an increase in the rate of effective donors compared to the same period in 2019, especially due to the sharp decrease in the family rejection rate, which was below 40% for the first time.^2^

Analysis of pre-pandemic data showed that between 1995 and 2015, Brazil averaged 20 kidney transplants per million population per year. The absolute number of kidney transplants during this period grew by 308%.^3^ Between 2015 and 2019, the country continued to perform well in kidney and other solid organ transplantation, ranking prominently in the world. Therefore, the question underlying this study was: how has the COVID-19 pandemic affected kidney transplant activity in Brazil in 2020?

## OBJECTIVE

To compare the kidney transplantation activities registered in Brazil between the pandemic (2020) and pre-pandemic (2019) periods.

## METHODS

This is a descriptive study conducted in March 2021. The annual reports of the Brazilian Transplantation Registry for 2019 and 2020 were included in this study. Data from the Brazilian Transplantation Registry are compiled by the Brazilian Organ Transplantation Society,^4^ which analyzes and publishes various information on national transplant activity in Brazil on a quarterly basis. The figures were reported to the Brazilian Organ Transplantation Society by the hospitals that performed the transplants. In this sense, the demand for kidney transplantation in the Brazilian Transplantation Registry is also presented as raw data calculated on the basis of the information provided by each service. Currently, there are approximately 160 kidney transplant centers in Brazil, distributed among the five geographic regions of the country. All data are presented in the Brazilian Transplant Registry as raw data based on information provided by individual facilities. Therefore, the number of facilities submitting data to the Brazilian Organ Transplantation Society may vary from year to year, justifying possible differences in the sum of data in each year.

### Data collection

Numerical information was extracted from the following variables: annual kidney transplantation, the number of patients already enrolled, added and deceased on the kidney transplant waiting list; number of kidney transplant teams. Data on the incidence of COVID-19 in Brazil at the end of 2020 were obtained from the official epidemiological report released by the Ministry of Health.^5^ Data were collected using a standardized form and subsequently tabulated in a Microsoft Excel spreadsheet.

Kidney transplant data were stratified considering donor type (living and deceased donors) and geographical region. The kidney transplant waiting list was stratified considering the general number and number of deaths on the waiting list. The number of kidney transplant teams includes all teams that performed at least one kidney transplant each year.

### Statistical analysis

All statistical analyzes were performed in the Microsoft Excel, version 2010, and we present descriptive data for all variables of interest. Data from the pandemic (2020) and pre-pandemic (2019) periods were compared by calculating the percentage difference between the years of interest.

### Ethical approval

We analyzed data from the Brazilian Transplantation Registry, which was made publicly available by the Brazilian Organ Transplantation Society. since we used publicly available data, it was not necessary to obtain formal consent for developing the study because the data were publicly available, patients were not identified, and sources were accurately cited.

## RESULTS

### Kidney transplantation activities

**Figure 1:**
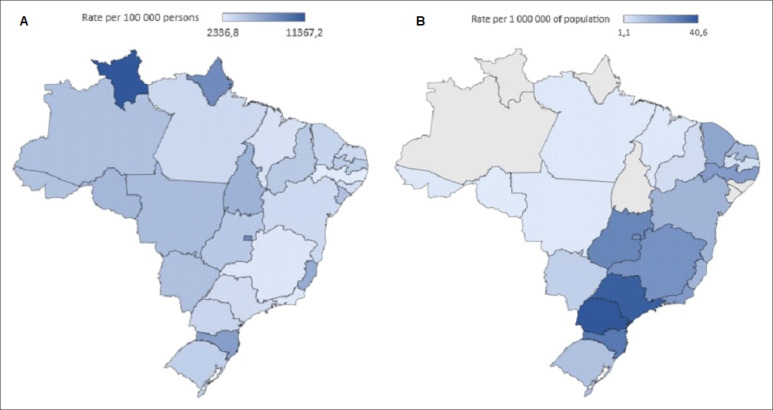
Regional rate in coronavirus disease 2019 (COVID-19) cases, (A) and kidney transplantation (KT) procedures; (B) during pandemic period (2020).

During the pandemic and pre-pandemic periods, 22.9 and 30.1 kidney transplants per million population were performed, respectively, a 23.9% decrease from the COVID-19 era. The largest decreases occurred in living donor kidney transplants (-58.8%) and in the Northern region (-79.5%) (Table 1). The percentage difference between kidney transplant volume and estimated demand was higher in the pandemic period (61.8%) than in the pre-pandemic period (49.8%) (Table 2). Analyzing data from each Brazilian state, we found that the decrease in kidney transplantation procedures was consonant with the increase in the incidence of COVID-19 (Figure 1).

**Table 1:** Kidney transplant* in the pandemic and pre-pandemic period, by donor type and region

Kidney transplant		Pandemic period (2020)	Pre-pandemic period (2019)	Percentual difference
**Donor type**				
	Living	2.1	5.1	−58.8
	Deceased	25.0	20.8	+20.2
**Region**				
	South	34.7	46.6	−25.5
	Southeast	30.9	38.1	−18.9
	Midwest	18.7	20.3	−7.8
	North	0.9	4.4	−79.5
	Northeast	12.5	20.2	−38.1
	Brazil	22.9	30.1	−23.9

* Expressed by 1 000 000 of population.

**Source:** Brazilian Transplantation Registry/Brazilian Organ Transplantation Society.

### Waiting list and transplant teams

**Figure 2:**
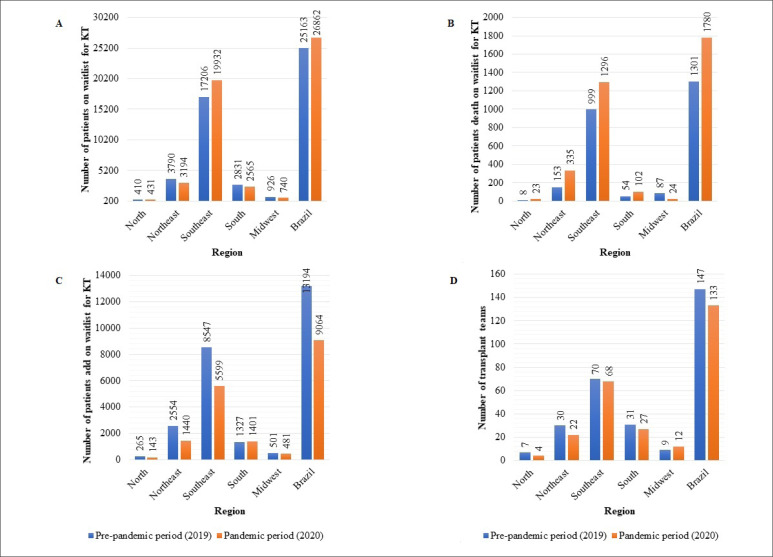
Number of patients on waitlist (A), that died on waitlist (B) and add (C) on kidney transplantation (KT) waitlist and number of transplant teams (D) in Brazil, according to the period of evaluation.

During the pandemic period (2020), there were 26,862 patients on the kidney transplant waiting list, compared with 25,163 during the pre-pandemic period, an increase of 6.8% (Figure 2A). The number of patients newly added to the waiting list decreased by 31.3% and was 9,064 in the pandemic period and 13,194 in the pre-pandemic period (Figure 2B). The number of patients who died on the waiting list before receiving a kidney transplant increased by 36.8% and to 1,780 in the pandemic period and 1,301 in the pre-pandemic period (Figure 2C). The number of transplant teams was 133 during the pandemic period and 147 in the pre-pandemic period, a decrease of 9.5% (Figure 2D).

## DISCUSSION

Considering the growth curve of the number of COVID-19 cases in June 2020, it is likely that the pandemic in Brazil is far from over and could break out again with a heterogeneous geographic distribution, given that the country is the size of a continent. It is worth noting that despite the pandemic, there are still other diseases, such as chronic kidney disease, that maintain their natural course and tend to more severe stages. According to the Brazilian Dialysis Census, the number of patients requiring renal replacement therapy has increased significantly recently.^6^ Kidney transplantation can improve life expectancy and quality of life for patients with chronic kidney disease, but the COVID-19 outbreak has had a significant impact on the kidney transplant activity in Brazil.

As shown in Table 1, the number of kidney transplants from living donors decreased significantly (-58%), the national rate decreased from 30.1 to 22.9 transplants per million population (-23.9%), and the North was the region the greatest negative impact (-79.5%). It is noteworthy that the North region already had low transplant activity in previous years due to the scarcity of available resources. With the outbreak of the COVID-19 pandemic, the numbers decreased even further as the region suffered from the severe impact of the pandemic on its population.

Alternatively, the South and Southeast regions already have a higher base volume and have more active services, including exclusive hospitals for transplantation, which have not become an open door for patients with COVID-19, as in the case of Hospital do Rim in São Paulo. In the Northeast region, there are few services, but they are very active. However, the transplant centers in the region are located in general hospitals, but due to the progression of the pandemic, these hospitals have become reference services for the care of patients with COVID-19.

This negative outlook has also been observed in other countries,^7^ such as the United Kingdom, where the kidney transplant rate has decreased by 68%^8^ and an estimated 1,670 kidney transplant opportunities have been lost, which will mean an increase in the number of patients in renal replacement therapy and on the waiting list.^9^ In the United States, kidney transplantation by living and deceased donors has decreased to 24% and 87%, respectively.^10^

A study conducted in Italy found that the number of kidney transplants at the beginning of the outbreak COVID-19 remained stable compared with previous years.^11^ In France, the decrease in deceased donor transplants was 90.6%.^12^ In Spain, 49 (76.6%) of 64 kidney transplant centers suspended activities, and 9.4% imposed restrictions during COVID-19.^13^ Kidney transplant rates also decreased significantly in Japan, Canada, Austria, the Netherlands, and Switzerland after the COVID-19 outbreak.^7^ An international survey found that 75% of Latin American kidney transplant centers stopped seeking living donor candidates after the COVID-19 pandemic.^14^ As the data show, the percentage decrease in the number of kidney transplants in Brazil (-23.9%) was acceptable compared with other developed countries, confirming the robustness of the Brazilian National Transplant Program.

Among the aspects that prevented the maintenance of kidney transplantation rates during the pandemic were the necessary distancing and social isolation, which made it difficult for patients to access health professionals and services; the lack of inpatient beds, especially in intensive care units; difficulties in the logistics of transporting organs from the collection facility to the kidney transplant center; the lack of human, material, and financial resources more focused on treating patients infected with COVID-19; and the collapse of health services, particularly in low- and middle-income countries.

The number of deaths from traumatic causes decreased, which also contributed to the reduction in donor numbers. Hospitals were over-extended with COVID-19 patients and many professionals were directed to the front line, which impacted communication to potential donors. Many transplant hospitals have become health services dedicated to patients with COVID-19, reducing and even suspending transplant activities for prolonged periods, in the interest of patient protection.

As shown in Table 2, the COVID-19 pandemic increased the percentage difference between the demand and supply of kidneys for transplantation. However, being a country with a large geographic area, the decline in kidney transplantation in Brazil shows considerable variation among the different regions of the country (Table 1). The decline in kidney transplantation rates showed a geographic distribution related to the increase in COVID-19 contamination rates (Figure 1). The availability of resources in a region directly impacts kidney transplantation rates, as this is a factor that has contributed to the suspension or reduction in activity.^7,14^ Additionally, the risk of contamination of recipients with COVID-19 virus is one of the most important clinical problems and a reason for the suspension of living donor transplants and the restriction of deceased donor transplants to be performed only after rigorous epidemiologic screening, according to the original recommendations of the American Society of Transplantation.^15^

**Table 2:** Kidney transplant volume and estimated demand, according to the period of report

Period	Kidney transplants	Estimated demand	Percentual difference
Pandemic (2020)	4,805	12,609	61.8
Pre-pandemic (2019)	6,283	12,510	49.8

**Source:** Brazilian Transplantation Registry/Brazilian Organ Transplantation Society.

For Brazilian kidney transplant programs, the recommendation of the Brazilian Organ Transplantation Society is that the donor-transplant process not be suspended as much as possible for the following reasons: 1) patients in need of a liver, heart, or lung will have their lives saved by a transplant, whose death is inevitable if the procedure is not performed within weeks or months; 2) in Brazil, an average of 6,000 kidney transplants are performed per year; for this reason, suspension of these procedures would lead to an increase in patients needing renal replacement therapy and an increase in the number of deaths of patients on the waiting list; 3) corneal and pancreatic transplants can be expanded, but not completely eliminated, as they are crucial in certain situations; 4) organ harvesting must be maintained in compliance with the rules of the Brazilian Organ Transplantation Society infection committee, with regard to the necessary care to avoid contamination by COVID-19; 5) if there are restrictions in performing transplants in a particular hospital, it is recommended to refer patients or even organs to the nearest service that can perform the procedure.^16^

The recommendations of the Brazilian Organ Transplantation Society for Brazilian programs to maintain kidney transplantation activity are plausible and defensible. During the pandemic period, the number of patients on the waiting list increased (Figure 2A), but the number of new patients added to the waiting list decreased (Figure 2B). These data are consistent with other studies conducted worldwide, and several countries have implemented strategies to minimize the impact of COVID-19 on kidney transplant program activity.^7,14^

The inactivity of the waiting list may have an impact on the congestion of Brazilian dialysis centers in the period after COVID-19, as is likely to be the case in other countries.^7^ Despite global concern about infection of kidney transplant recipients with the SARS Cov-2 virus, a study conducted in the United States has shown that patients on the waiting list are at significantly higher risk of hospitalization and death compared with kidney transplant recipients.^17^ In the United Kingdom, 10% of patients on the waiting list and with COVID-19 died.^18^ In France, 42% of deaths of patients on the waiting list were attributable to COVID-19.^19^ We cannot retrieve specific Brazilian data; however, we verified that the number of deaths on the waiting list increased during the pandemic period (Figure 2C). The most recent report from the Brazilian Organ Transplantation Society found that the deaths among patients on the kidney transplant waiting list increased by 40% in the first quarter of 2021 compared with the same period last year.^20^

Another negative finding was the decrease in the number of transplant teams (Figure 2D). One of the possible reasons for this may have been the need to redirect health professionals to the front line in treating patients infected with COVID-19. The delay in initiating containment measures, and especially the delay in vaccinating the population, had devastating consequences for the public health system in our country.^21^ The fact that the Brazilian government considered SARS-Cov-2 infection as just another common infection at the beginning of the outbreak and delayed the national response to the pandemic may have contributed much to the inexorable increase in the number of deaths and cases of COVID-19 in the country.

Despite the political negligence, the Transplant Infections Committee of the Brazilian Organ Transplantation Society has presented several recommendations for Brazilian health services performing kidney transplants, including Donor management (with the epidemiological investigation of suspected or confirmed cases of SARS-CoV-2; clinical investigation of respiratory symptoms; laboratory investigation with reverse transcription-polymerase chain reaction test for SARS-CoV-2 of respiratory samples, depending on the availability of the test); Establishment of criteria for acceptance of organs (no recommendation to use organs from donors with active COVID-19); criteria for evaluation of kidney transplant candidates (perform only one emergency transplant in a candidate with active COVID-19, candidates with active COVID-19 are cleared for transplant 28 days after infection).^22^ As in Brazil, the United States,^10^ Canada,^23^ Spain,^13^ Italy,^11^ the United Kingdom,^9^ and the Netherlands^24^ implemented good organ donation and transplantation practices during the COVID-19 pandemic.

### Future perspectives

The Brazilian Transplant System is an international reference, mainly because of the financial subsidy of almost all transplants by the public health system.^25^ However, looking to the post-COVID-19 era, we believe that Brazil will have a gradual, slow, and regionalized recovery, both in terms of the good kidney transplant rates that the country had in the pre-pandemic period and in several other areas. The inequality of income and resources among Brazilian regions was even greater during COVID-19, and integration among the system, health services, and society will be essential for the country's recovery.

## CONCLUSION

The COVID-19 pandemic has drastically affected Brazil and had a significant negative impact on kidney transplant activity in the country. This is the first study to analyze national data on kidney transplant activity in the first year of the pandemic and compare it with the previous period. The results may help policymakers and transplant center managers implement strategies to help the Brazilian National Transplant System regain its world-leading position.
